# Trends in Exclusive Non-Cigarette Tobacco Smoking in England: A Population Survey 2013–2023

**DOI:** 10.1093/ntr/ntae021

**Published:** 2024-03-05

**Authors:** Sarah E Jackson, Lion Shahab, Jamie Brown

**Affiliations:** Department of Behavioural Science and Health, University College London, London, UK; SPECTRUM Consortium, Edinburgh, UK; Department of Behavioural Science and Health, University College London, London, UK; SPECTRUM Consortium, Edinburgh, UK; Department of Behavioural Science and Health, University College London, London, UK; SPECTRUM Consortium, Edinburgh, UK

## Abstract

**Introduction:**

The UK Government intends to implement a “smokefree generation” policy prohibiting the sale of all tobacco products to people born after 2008. National surveys provide comprehensive data on cigarette smoking, but little is known about patterns of non-cigarette tobacco smoking across key population groups.

**Aims and Methods:**

Using data from a nationally representative cross-sectional survey of adults in England, collected monthly between September 2013 and September 2023 (*n* = 196 721), we estimated time trends in exclusive non-cigarette tobacco (eg, cigar/pipe/shisha) smoking prevalence, overall and by age, gender, occupational social grade, region, ethnicity, and vaping status. Interviews were conducted face-to-face until March 2020 and via telephone thereafter.

**Results:**

From September 2013 to September 2023, there was a non-linear increase in exclusive non-cigarette tobacco smoking prevalence (from 0.36% to 1.68%; prevalence ratio = 4.72 [95% CI = 3.43–6.48]). Prevalence was relatively stable up to February 2020 (at an average of 0.46%), then increased sharply at the start of the COVID-19 pandemic (at the same time as survey methods changed), to 0.90% (0.82%–0.99%) in March 2020. This was followed by a steadier rise, peaking at 1.97% in May 2022, before falling slightly to 1.68% by September 2023. In 2022/2023, 1 in 10 smokers (10.8% [9.64%–12.0%]) exclusively used non-cigarette tobacco. The rise in prevalence was observed across all subgroups but was most pronounced among younger adults (eg, reaching 3.21% of 18-year-olds vs. 1.09% of 65-year-olds). Prevalence was consistently higher among men and current vapers.

**Conclusions:**

Although exclusive use of non-cigarette combustible tobacco remains rare among adults in England, it has increased in recent years, particularly among younger ages. As of September 2023, there were approximately 772 800 adult exclusive non-cigarette tobacco smokers in England; around five times more than a decade earlier.

**Implications:**

The proportion of adults in England who do not use cigarettes at all but smoke other combustible tobacco products has increased substantially in recent years, with a particularly pronounced rise among young people. The inclusion of non-cigarette combustible tobacco products under the proposed “smokefree generation” policy is therefore likely to be important for achieving the greatest reduction in youth uptake of tobacco smoking, as it would ensure young people who are unable to legally buy cigarettes do not buy other combustible tobacco products that are similarly harmful to health.

## Introduction

In October 2023, the UK Government announced its intention to implement a “smokefree generation” policy in England, which would progressively increase the age of sale of tobacco products such that anyone born on or after 1 January 2009 would never legally be able to buy tobacco.^1^ Non-cigarette tobacco products would be included under this proposed policy^1^; in England, the most commonly used non-cigarette combustible tobacco products are cigars, closely followed by waterpipes (shisha), pipes, and cigarillos.^2^

There is good evidence from representative population surveys on the prevalence and patterns of cigarette smoking in England,^3^ providing a clear picture of who would be targeted by this policy. Such surveys suggest the exclusive use of other non-cigarette combustible products was relatively rare in England through 2015,^4^ but little is known about the latest numbers of who would be affected by the new policy. It is particularly relevant to assess the latest data because prevalence may have changed substantially in the context of a rapidly evolving nicotine market, the Coronavirus disease-2019 (COVID-19) pandemic, and other major socioeconomic shifts in the last five years.

Understanding how prevalent exclusive non-cigarette tobacco smoking is, and how this is changing over time, is important for monitoring purposes and for informing and evaluating policy. The Smoking Toolkit Study (a representative, cross-sectional survey) collects data on exclusive non-cigarette tobacco smoking among adults in England each month. This study aimed to use data collected over the past decade to address the following research questions:

1. What is the overall prevalence of exclusive non-cigarette tobacco smoking among adults in England, and how does this vary by sociodemographic characteristics and vaping status?
2. How has the prevalence of exclusive non-cigarette tobacco smoking changed between 2013 and 2023?
3. Have trends over this period varied by sociodemographic characteristics and vaping status?

## Method

### Pre-registration

The study protocol and analysis plan were pre-registered on Open Science Framework (https://osf.io/w7f3m/). In addition to our planned analyses, we calculated the absolute and relative change in the prevalence of exclusive non-cigarette tobacco smoking across the whole time series and estimated the number of adult exclusive non-cigarette tobacco smokers in England in September 2013 and September 2023 (see *Statistical Analysis* section for details). We also added an unplanned segmented regression analysis to examine whether the start of the COVID-19 pandemic (and change in the mode of data collection, from face-to-face to telephone interviews) was associated with a step-level change in exclusive non-cigarette tobacco smoking and a change in trend.

### Design

Data were drawn from the ongoing Smoking Toolkit Study, a monthly cross-sectional survey of a representative sample of adults aged ≥16 years in England.^5^ The study uses a hybrid of random probability and simple quota sampling to select a new sample of approximately 1700 adults each month. Comparisons with sales data and other national surveys indicate that key variables including sociodemographic characteristics, smoking prevalence, and cigarette consumption are nationally representative.^5,6^

We used data collected from September 2013 through September 2023 (the most recent data at the time of analysis). Data were initially collected through face-to-face computer-assisted interviews. However, social distancing restrictions under the COVID-19 pandemic meant no data were collected in March 2020 and data from April 2020 onward were collected via telephone. The telephone-based data collection used broadly the same combination of random location and quota sampling, and weighting approach as the face-to-face interviews and comparisons of the two data collection modalities indicate good comparability.^7–9^

### Population

We used data from adults aged ≥18 years. Because data were not collected from 16- to 17-year-olds between April 2020 and December 2021, we restricted our sample to those aged ≥18 for consistency across the time series.

## Measures

Smoking status was assessed by asking participants which of the following best applies to them:

1. I smoke cigarettes (including hand-rolled) every day.
2. I smoke cigarettes (including hand-rolled), but not every day.
3. I do not smoke cigarettes at all, but I do smoke tobacco of some kind (eg, pipe, cigar, or shisha).
4. I have stopped smoking completely in the last year.
5. I stopped smoking completely more than a year ago.
6. I have never been a smoker (ie, smoked for a year or more).

Those who responded *a*–*c* were considered current smokers. Those who responded *c* were considered exclusive non-cigarette tobacco smokers and those who responded *a* or *b* cigarette smokers.

Age was modeled as a continuous variable using restricted cubic splines (see *StatisticalAnalysis* section). We also provided descriptive data by age group (18–24, 25–34, 35–44, 45–54, 55–64, or ≥65).

Gender was self-reported as man or woman. In more recent waves, participants have also had the option to describe their gender in another way; those who identified in another way were excluded from analyses by gender due to low numbers.

The occupational social grade was categorized as ABC1 (includes managerial, professional, and upper supervisory occupations) and C2DE (includes manual routine, semi-routine, lower supervisory, and long-term unemployed).

Region in England was categorized as North, Midlands, and South.

Ethnicity was categorized as White and minority ethnic groups. Data on ethnicity were not collected between April and August 2020.

Vaping status was assessed with several questions asking about the use of a range of nicotine products. Current smokers were asked “Do you regularly use any of the following in situations when you are not allowed to smoke?”; current smokers and those who have quit in the past year were asked, “Can I check, are you using any of the following either to help you stop smoking, to help you cut down or for any other reason at all?”; and non-smokers were asked “Can I check, are you using any of the following?”. Those who reported using an e-cigarette in response to any of these questions were considered current vapers.

### Statistical Analysis

Analyses were done in R version 4.2.1. The Smoking Toolkit Study uses raking to weight the sample to match the population in England. This profile is determined each month by combining data from the UK Census, the Office for National Statistics mid-year estimates, and the annual National Readership Survey.^5^ The following analyses used weighted data. We excluded participants with missing data on smoking status. Missing cases on other variables (see Table 1 for details) were excluded on a per-analysis basis.

**Table 1.** Unadjusted Weighted Prevalence of Exclusive Non-tobacco Cigarette Smoking Among Adults in England: Data Aggregated Across the Study Period (September 2013–September 2023)

Characteristic	*N* ^*^	Prevalence, % [95% CI]^†^
All adults	196 721	0.88 [0.84–0.93]
Age (years)		
18-24	25 112	1.19 [1.04–1.34]
25–34	29 324	1.18 [1.05–1.32]
35–44	28 272	0.85 [0.73–0.97]
45–54	31 160	0.77 [0.67–0.87]
55–64	31 596	0.69 [0.59–0.79]
≥65	51 257	0.71 [0.64–0.79]
Gender		
Men	97 957	1.24 [1.16–1.32]
Women	98 115	0.52 [0.47–0.56]
Missing^‡^	649	—
Occupational social grade		
ABC1 (more advantaged)	118 174	0.85 [0.79–0.90]
C2DE (less advantaged)	78 547	0.92 [0.85–10.0]
Region in England		
North	57 888	0.77 [0.69–0.85]
Midlands	58 809	0.86 [0.78–0.95]
South	80 024	0.97 [0.90–1.04]
Minority ethnic group^§^		
No	159 768	0.79 [0.74–0.83]
Yes	27 654	1.25 [1.10–1.40]
Missing^§^	9299	—
Current vaping		
No	184 577	0.71 [0.67–0.76]
Yes	12 144	3.27 [2.92–3.61]

^*^Unweighted sample size.

^†^Weighted proportion of participants surveyed between September 2013 and September 2023 reporting exclusive non-cigarette tobacco smoking.

^‡^Includes 513 participants who described their gender in another way; prevalence of exclusive non-cigarette tobacco smoking among these participants was 3.87% [95% CI = 2.20–5.54].

^§^Ethnicity was not assessed in certain waves (April–August 2020). Missing cases include participants surveyed in these waves.

We reported the prevalence (with 95% confidence interval [CI]) of exclusive non-cigarette tobacco smoking, overall (aggregated across survey waves) and by survey year, among all adults and by age, gender, occupational social grade, region in England, ethnicity, and vaping status. For context, we also provided descriptive data on the proportion of smokers using cigarettes vs. exclusively using non-cigarette combustible tobacco by survey year.

Trends in exclusive non-cigarette tobacco smoking prevalence over the study period were analyzed using logistic regression with exclusive non-cigarette tobacco smoking as the outcome and time (survey month) modeled using restricted cubic splines with five knots (sufficient to accurately model trends across years without overfitting). This allowed for flexible and non-linear changes over time while avoiding categorization. We estimated the total number of adults in England exclusively smoking non-cigarette tobacco in September 2023 based on the most recent (2021) mid-year population estimates for England,^10^ and in September 2013 based on 2013 mid-year population estimates for England.^11^

To explore moderation of trends by age, gender, occupational social grade, region in England, ethnicity, and vaping status, we repeated the models including the interaction between the moderator of interest and time—thus allowing for time trends to differ across sub-groups. Each of the interactions was tested in a separate model. Age was modeled using restricted cubic splines with three knots (placed at the 5%, 50%, and 95% quantiles), to allow for a non-linear relationship between age and exclusive non-cigarette tobacco smoking.

We used predicted estimates from these models to plot the prevalence of exclusive non-cigarette tobacco smoking over the study period, among all adults and within each subgroup of interest. As age was modeled continuously, we displayed estimates for six specific ages (18-, 25-, 35-, 45-, 55-, and 65-year-olds) to illustrate how trends differ across ages. Note that the model used to derive these estimates included data from participants of all ages, not only those aged exactly 18, 25, 35, 45, 55, or 65 years. We also reported the absolute and relative changes across the whole time series. Absolute changes were calculated as the percentage point change in prevalence (prevalence in September 2023 minus prevalence in September 2013) and relative changes were calculated as the prevalence ratio (PR; prevalence in September 2023 divided by prevalence in September 2013). We presented these alongside 95% CIs calculated using bootstrapping.

In an unplanned analysis, we used segmented regression to assess whether there was a step change in exclusive non-cigarette tobacco smoking at the start of the COVID-19 pandemic, which also coincided with the change from face-to-face to telephone interviews. We used logistic regression to model the trend in exclusive non-cigarette tobacco smoking before the pandemic (underlying secular trend; coded 1 … *n*, where *n* was the total number of waves), the step-level change (coded 0 before the start of the pandemic in March 2020 and 1 after), and change in the trend (slope) post-onset of the pandemic relative to pre-pandemic (coded 0 before the pandemic and 1 … *m* from April 2020 onward, where *m* was the number of waves after the start of the pandemic). A linear pre-pandemic and pandemic trend was assumed, based on the relatively short length of the time series (meaning we expected negligible differences between log-linear and linear trends). We used predicted estimates from these models to plot time trends in the prevalence of exclusive non-cigarette tobacco smoking alongside unmodeled monthly data points.

## Results

A total of 197 299 (unweighted) adults aged ≥18 years were surveyed between September 2013 and September 2023. We excluded 578 (0.3%) with missing data on smoking status, leaving a final sample for analysis of 196 721 participants (weighted mean [*SD*] age = 47.9 [18.6] years; 50.8% women).

### Overall Estimates of Prevalence

Across the study period, the overall prevalence of exclusive non-cigarette tobacco smoking was 0.88% (Table 1). Groups with notably higher overall prevalence included younger adults, men and non-binary people, minority ethnic groups, and current vapers (Table 1). However, these overall estimates masked different patterns over time between subgroups of the population.

### Time trends

From September 2013 to September 2023, our primary (pre-registered) model indicated that the prevalence of exclusive non-cigarette tobacco smoking among all adults increased from 0.36% to 1.68% (PR = 4.72 [95% CI = 3.43–6.48]; Table 2). This equates to approximately 772 800 adult exclusive non-cigarette tobacco smokers in England in September 2023 (46 million adults^10^ × 1.68%); up from approximately 151 200 in September 2013 (42 million adults^11^ × 0.36%). The proportion of adult smokers in England who exclusively smoke non-cigarette tobacco in 2022/23 was significantly higher than it was a decade earlier (10.8% [9.64%–12.0%] in 2022/23 vs. 2.25% [1.76%–2.73%] in 2013/2014; Figure S1).

**Table 2.** Modeled Estimates of the Change in Prevalence of Exclusive Non-cigarette Tobacco Smoking Among Adults in England from September 2013 to September 2023

	Prevalence, % (95% CI)^*^		Change in prevalence	
Characteristic	September 2013	September 2023	Relative change, prevalence ratio (95% CI)^†^	Absolute change, percentage points (95% CI)^‡^
All adults	0.36 [0.27–0.48]	1.68 [1.45–1.94]	4.72 [3.43–6.48]	1.32 [1.07–1.60]
Age (years)^§^				
18	0.19 [0.08–0.45]	3.21 [2.29–4.48]	16.7 [7.62–47.9]	3.02 [2.07–4.18]
25	0.24 [0.13–0.43]	2.61 [2.07–3.29]	11.1 [6.39–23.3]	2.38 [1.82–3.01]
35	0.31 [0.21–0.46]	1.97 [1.64–2.35]	6.37 [4.28–10.3]	1.66 [1.30–2.04]
45	0.38 [0.25–0.59]	1.53 [1.23–1.90]	3.98 [2.57–6.66]	1.14 [0.80–1.54]
55	0.44 [0.28–0.67]	1.26 [1.01–1.56]	2.87 [1.85–4.94]	0.82 [0.51–1.17]
65	0.46 [0.32–0.65]	1.09 [0.87–1.38]	2.39 [1.60–3.76]	0.64 [0.35–0.95]
Gender				
Men	0.69 [0.51–0.94]	2.17 [1.80–2.61]	3.13 [2.26–4.52]	1.48 [1.04–1.94]
Women	0.06 [0.03–0.13]	1.07 [0.83–1.38]	18.4 [9.06–52.5]	1.01 [0.77–1.30]
Occupational social grade				
ABC1 (more advantaged)	0.40 [0.27–0.60]	1.61 [1.35–1.92]	4.00 [2.75–6.36]	1.21 [0.94–1.52]
C2DE (less advantaged)	0.30 [0.19–0.46]	1.77 [1.39–2.26]	5.94 [3.74–9.85]	1.47 [1.06–1.95]
Region in England				
North	0.35 [0.22–0.56]	1.44 [1.11–1.88]	4.08 [2.78–8.99]	1.09 [0.78–1.71]
Midlands	0.36 [0.27–0.48]	1.64 [1.41–1.91]	4.60 [2.09–6.48]	1.28 [0.64–1.52]
South	0.36 [0.23–0.56]	1.86 [1.52–2.28]	5.18 [3.55–9.78]	1.50 [1.19–2.01]
Ethnicity				
White	0.34 [0.25–0.47]	1.67 [1.42–1.97]	4.88 [3.52–7.14]	1.33 [1.05–1.63]
Minority ethnic group	0.40 [0.18–0.91]	2.20 [1.59–3.04]	5.49 [2.64–15.1]	1.80 [1.08–2.62]
Using e-cigarettes				
No	0.32 [0.23–0.43]	1.25 [1.05–1.48]	3.95 [2.84–5.79]	0.93 [0.70–1.17]
Yes	1.25 [0.62–2.51]	4.71 [3.60–6.14]	3.76 [1.99–9.21]	3.46 [2.01–4.89]

CI = confidence interval.

Unmodeled estimates of prevalence within each survey year are provided in Supplementary Table S1.

^*^Data for September 2013 and September 2023 are weighted estimates of prevalence in these months (the first and last in the study period) from logistic regression with survey month modeled non-linearly using restricted cubic splines (five knots).

^†^Relative change in prevalence across the whole time series (calculated as prevalence in September 2023 divided by prevalence in September 2013) with 95% CI calculated using bootstrapping.

^‡^Absolute percentage point change in prevalence across the whole time series (calculated as prevalence in September 2023 minus prevalence in September 2013) with 95% CI calculated using bootstrapping.

^§^Note that the model used to derive these estimates included data from participants of all ages, not only those who were aged exactly 18, 25, 35, 45, 55, or 65 years.

**Figure 1.**
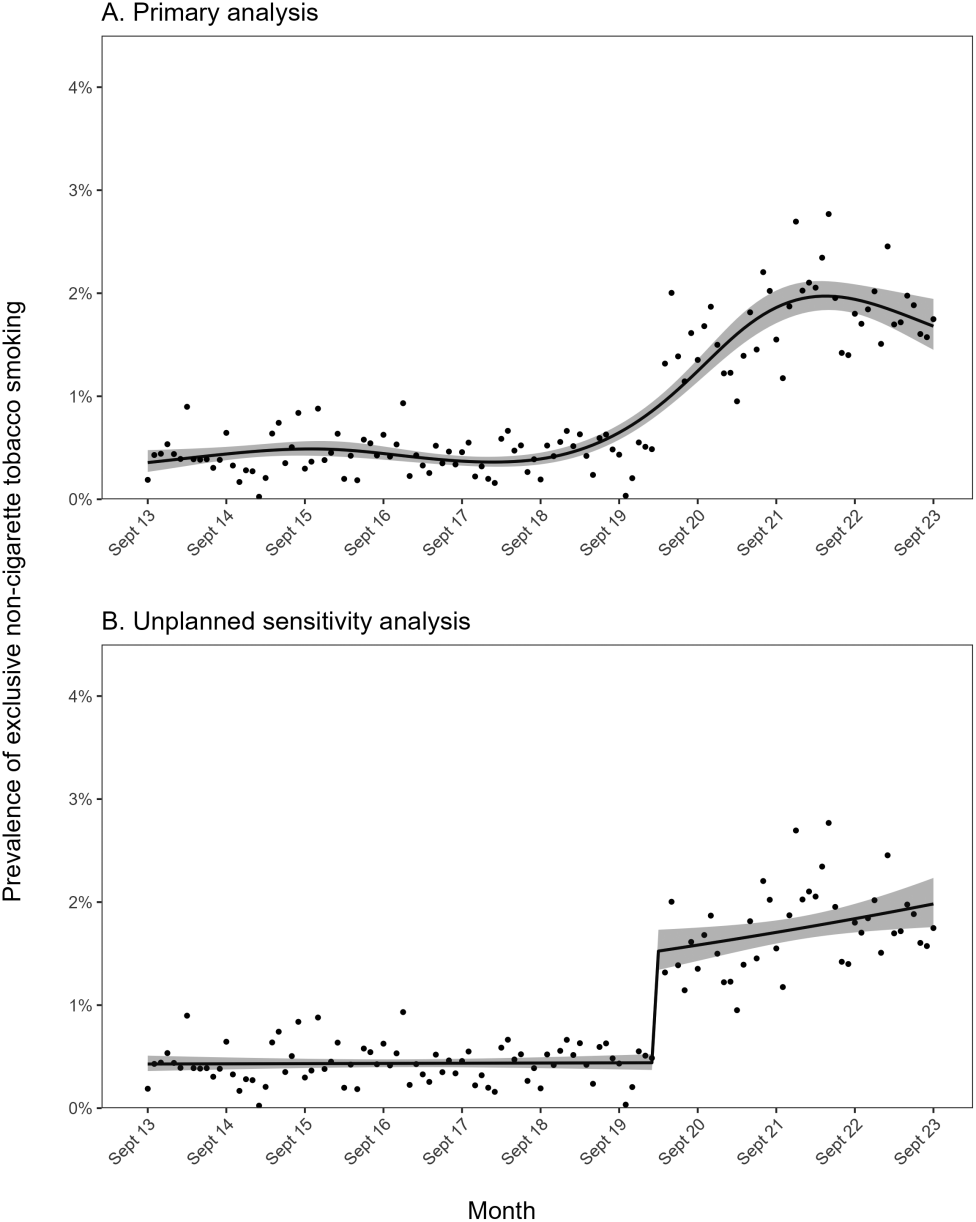
Trends in exclusive non-cigarette tobacco smoking among adults in England, September 2013 to September 2023. Panel A shows the results of the primary model, which modeled survey wave non-linearly using restricted cubic splines (five knots). Panel B shows the results of an unplanned sensitivity analysis, which used a segmented regression approach to model associations of the start of the COVID-19 pandemic with a step-level change in prevalence and a change in trend. Lines represent modeled weighted prevalence by monthly survey wave. Shaded bands represent 95% confidence intervals. Points represent unmodeled weighted prevalence by month.

The increase over time was not linear (Figure 1). Our primary model suggested prevalence was relatively stable up to February 2020, at an average of 0.46% (95% CI = 0.40%–0.53%), then increased sharply at the start of the COVID-19 pandemic, to 0.90% (0.82%–0.99%) in March 2020. This was followed by a steadier rise, peaking at 1.97% (1.84%–2.12%) in May 2022, before falling slightly to 1.68% (1.45%–1.94%) by September 2023 (Figure 1A).

We undertook an unplanned segmented regression analysis to explore the extent to which this pattern may have been driven by a step change at the start of the COVID-19 pandemic (March 2020), which also coincided with a change from face-to-face to telephone interviews, as opposed to the more gradual change in prevalence observed in our primary model. We observed a significant step-level increase in the prevalence of exclusive non-cigarette tobacco smoking at the start of the pandemic (OR_step-change_ = 3.475, 95% CI = 2.783–4.338), from 0.44% (95% CI = 0.37%–0.52%) in February 2020 to 1.52% (1.34%–1.73%) in March 2020 (Figure 1B), which may have at least partially reflected the change in data collection. However, there was also a notable change in trend (OR_Δtrend_ = 1.006, 95% CI = 1.000–1.013; Figure 1B). Before the pandemic, when data were collected face-to-face, the prevalence of exclusive non-cigarette tobacco smoking was stable (monthly trend: OR_trend_ = 1.000, 95% CI = 0.996–1.004). After the onset of the pandemic, when data were collected via telephone, the prevalence of exclusive non-cigarette tobacco smoking increased by 0.6% per month (OR_trend_ × OR_Δtrend_ = 1.000 × 1.006 = 1.006)—or 7.2% per year (0.6% × 12 months; note these percentages represent the relative rather than absolute percentage point increase). Prevalence increased from 1.52% (1.34%–1.73%) in March 2020 to 1.98% (1.76%–2.23%) in September 2023.

**Figure 2.**
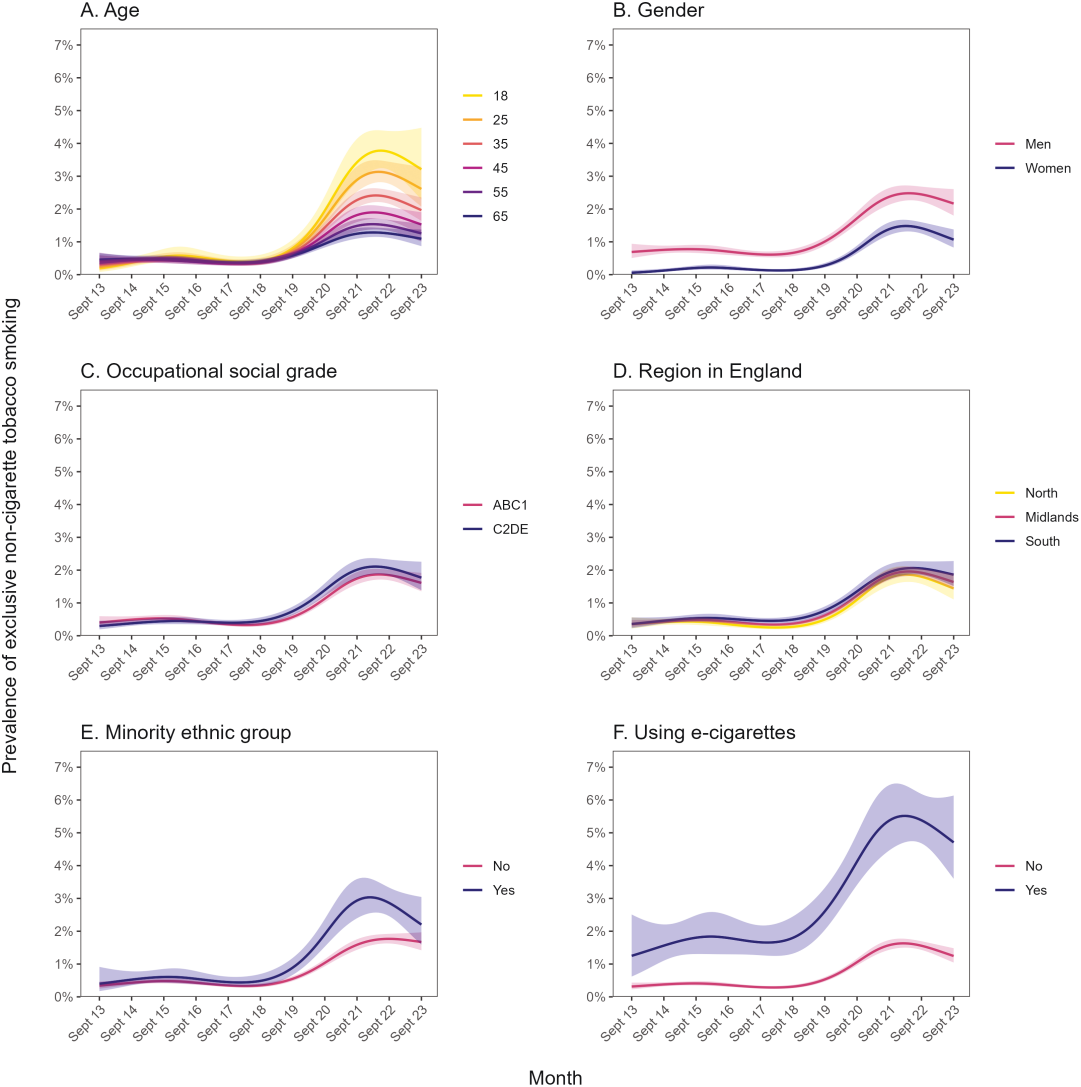
Trends in exclusive non-cigarette tobacco smoking within subgroups of adults in England, September 2013 to September 2023. Panels show trends by (**A**) age, (**B**) gender, (**C**) occupational social grade, (**D**) region in England, (**E**) ethnicity, and (**F**) vaping status. Lines represent modeled weighted prevalence by monthly survey wave, modeled non-linearly using restricted cubic splines (five knots). Shaded bands represent 95% confidence intervals.

This rise in the prevalence of exclusive non-cigarette tobacco smoking was observed across all population subgroups to varying degrees (Figure 2). In particular, there were substantial differences by age (Figure 2A). In September 2013, the prevalence of exclusive non-cigarette tobacco smoking was slightly lower at younger ages (eg, 0.19% among 18-year-olds compared with 0.46% among 65-year-olds; Table 2). However, this pattern reversed over the subsequent decade as prevalence grew more rapidly and to higher levels among younger than older adults. As a result, in September 2023 prevalence of exclusive non-cigarette tobacco smoking was significantly higher at younger ages (eg, 3.21% among 18-year-olds compared with 1.09% among 65-year-olds; Table 2).

Across the period, the prevalence of exclusive non-cigarette tobacco smoking was significantly higher among men than women (Table S1), but time trends did not differ substantially (Figure 2B). The relative increase in prevalence was larger among women (PR = 18.4 vs. 3.13 among men), who started from a very low baseline, but absolute changes over time were similar (Table 2).

Prevalence was similar across occupational social grades and regions in England, with no notable differences in changes over time (Figure 2C and D; Table 2).

The prevalence of exclusive non-cigarette tobacco smoking was similar across ethnic groups up to 2019, after which point patterns diverged (Figure 2E). The rise from 2020 onward was larger among minority ethnic groups, peaking at 3.03% (95% CI = 2.55–3.60) in February 2022 compared with a peak of 1.77% (95% CI = 1.63–1.92) in September 2022 among White participants. The gap in prevalence narrowed between February 2022 and September 2023, with prevalence falling to 2.20% (95% CI = 1.59–3.04) in September 2023 among minority ethnic groups but remaining relatively stable among White participants (1.67% [95% CI = 1.42–1.97] in September 2023). As a result, changes from the start to the end of the time series were similar across ethnic groups (Table 2).

Across the period, prevalence of exclusive non-cigarette tobacco smoking was significantly higher among vapers than non-vapers (Table S1). Although the absolute change in prevalence was larger among vapers (+3.46 vs. +0.93 percentage points), the relative increase was similar by vaping status (Table 2).

## Discussion

Between September 2013 and September 2023, there was a non-linear increase in the prevalence of exclusive non-cigarette tobacco smoking among adults in England. Although this rise in non-cigarette tobacco smoking was observed across all population subgroups, there was a significant age gradient whereby younger age was associated with a larger increase in prevalence. Across the decade, the prevalence was consistently higher among men than women (as has been documented in other surveys^2,4^) and among current vapers than non-vapers but was similar across occupational social grades and regions.

The timing of the rapid rise in prevalence of exclusive non-cigarette tobacco smoking we observed coincided with the onset of the COVID-19 pandemic in March 2020. It is possible this was driven by concerns about the health risks of smoking cigarettes. Early in the pandemic, there were concerns and studies suggesting that smoking might be associated with a higher risk of severe COVID-19 outcomes.^12–14^ Some people may have chosen to switch to non-cigarette combustible tobacco products, thinking that they could be less harmful or reduce their health risks.^15^ Consistent with this theory, there was a surge in quitting activity among smokers in England at the start of the pandemic,^7,8^ and cross-sectional data from the International Tobacco Control (ITC) Four Country Survey collected between February and June 2020 showed that a substantial proportion (11.8%) of recent ex-cigarette smokers in England, the United States, Canada, and Australia were using other combustible tobacco at this time.^2^

Another possible driver may have been economic factors. Many people have faced financial challenges due to the economic impact of the pandemic.^16^ Non-cigarette combustible tobacco products may have been perceived as more affordable options compared with traditional cigarettes, leading some people to switch to these alternatives. Alternatively, it is possible that a shift from in-person to online tobacco purchasing made consumers aware of the wider range of available products, or that home working was better suited to consumption of non-cigarette products or avoided concerns around social norms towards using them. It is also possible that some participants reporting non-cigarette tobacco use were referring to cannabis; there is evidence cannabis use may have increased during the pandemic.^17^

Besides the pandemic, another factor that may have driven the rise in exclusive use of non-cigarette combustible tobacco was the UK’s ban on menthol cigarettes in May 2020.^18^ The ban does not apply to non-cigarette combustible tobacco products and the tobacco industry has responded by launching new products to the market that bypass the legislation (eg, cigarillos with menthol flavor capsules that closely resemble cigarettes).^19^ It is possible that people who had been smoking menthol cigarettes before the ban switched to these non-cigarette menthol products when their usual product of choice was no longer available. However, given the close resemblance of these cigarillos to cigarettes,^19^ it is also possible that some people who use these products may consider them to be cigarettes and continue to report being cigarette smokers in response to the question assessing smoking status.

Concern for health as a motive for (switching to) non-cigarette tobacco smoking might be supported by the differences in the pattern of results we observed across ethnic groups. In the early stages of the pandemic, there was a particularly pronounced rise in exclusive non-cigarette tobacco smoking among minority ethnic groups, many of whom were at increased risk of severe COVID-19 outcomes.^20^ After the vaccination program was rolled out and these risks were reduced, the prevalence of exclusive non-cigarette tobacco smoking among minority ethnic groups fell to levels comparable with White adults.

However, the rise in exclusive non-cigarette tobacco smoking since 2020 was also more pronounced among younger than older ages. This is the opposite pattern to what might be expected if the rise was being driven by concerns for health, given risks of adverse COVID-19 outcomes were greater at older ages.^21^ Cross-sectional data from the ITC Four Country Survey also documented higher levels of use of non-cigarette tobacco products among younger than older age groups in 2020.^2^ This pattern of results may reflect greater exploration of different products among younger adults.^2^ Over the same period when exclusive non-cigarette tobacco smoking increased, there was also a marked increase in vaping among adolescents and young adults,^22–24^ which may have prompted experimentation with other nicotine products. Across the study period, prevalence of exclusive non-cigarette tobacco smoking was higher among people who vaped. We did not capture the frequency of product use, so our data do not provide any insight into patterns of use (ie, regular daily use vs. experimental use) among those reporting exclusive non-cigarette tobacco smoking. Further research is needed to better understand the reasons exclusive non-cigarette tobacco smoking has increased since the start of the pandemic, the specific products being used, patterns of use, and how these differ across population subgroups.

Our findings have implications for policy. The UK Government plans to introduce a “smokefree generation” policy, which would prohibit the sale of tobacco products in England to anyone born on or after January 1, 2009.^1^ Our data indicate that although cigarettes remain the product of choice for the vast majority of adult smokers in England, exclusive use of other combustible tobacco products (such as cigars, pipes, or shisha) has risen in recent years. As of September 2023, we estimate that approximately 772 800 adults in England (more than 1 in every 10 smokers) do not use cigarettes at all but smoke other combustible tobacco products—five times more than the estimated number in September 2013 (approximately 151 200). The rise has been even more pronounced among younger ages. Collectively, these results suggest that while it is much less common than cigarette smoking, exclusive non-cigarette tobacco smoking is not negligible and possibly rising. The inclusion of these products in a smokefree generation policy is likely to be important for achieving the greatest reduction in youth uptake of tobacco smoking, as it would ensure young people who are unable to legally buy cigarettes do not buy other combustible tobacco products that are similarly harmful to health.^25,26^

Strengths of this study include the large, nationally representative sample and monthly data collection, which provides granular and up-to-date estimates of prevalence. There were also limitations. First, there were limitations with the item used to measure smoking status. The first three response options provided for this question are not mutually exclusive (ie, people can be a user of both cigarettes and non-cigarette tobacco), but participants could only select one option. As a result, tobacco users who consume both cigarettes and other forms of smoked tobacco concurrently were classified as cigarette smokers. Our study was therefore only able to provide evidence of the prevalence of *exclusive* non-cigarette tobacco smoking. As such, we cannot tell from our data whether there has been an overall rise in the prevalence of *any* non-cigarette tobacco smoking, or whether the rise in exclusive use was the result of changing patterns of dual use. For example, if the prevalence of dual use of non-cigarette combustible tobacco cigarettes has been declining alongside the rising prevalence of exclusive use of non-cigarette combustible tobacco, then the overall trend in the prevalence of any non-cigarette tobacco smoking could be stable (or even decreasing). Nonetheless, the prevalence of exclusive non-cigarette tobacco smoking remains important, as any dual use with cigarettes is already captured within estimates of cigarette smoking whereas an increase in exclusive non-cigarette tobacco smoking may go undetected by other major surveys (which tend to only ask about cigarette smoking). Another limitation of the survey measures is that the type of tobacco products smoked was not assessed, so we were unable to determine whether the recent rise in exclusive non-cigarette tobacco smoking was driven by a particular product category. In addition, use of non-cigarette tobacco products was self-reported and there was potential for participants to misinterpret what is meant by “smoke tobacco of some kind.” Although the question specifically stated that it was not referring to e-cigarettes, it is possible that the higher prevalence of exclusive non-cigarette tobacco use among current vapers partly reflected people misinterpreting the question to include e-cigarette use. Likewise, it is plausible that some participants may have considered cannabis use as another form of tobacco smoking. Further research is required to provide insight into changes in the prevalence of any non-cigarette combustible tobacco use and the specific products driving the overall rise in exclusive use.

Another key limitation was the change in the mode of data collection (from face-to-face to telephone interviews) in April 2020 when the COVID-19 pandemic started, which coincided with the sharp rise in the prevalence of exclusive non-cigarette tobacco smoking we observed. Previous studies have shown that estimates of substance use behavior tend to be very similar across face-to-face and telephone interviews.^27–29^ Consistent with this, we collected data in parallel telephone and face-to-face surveys in March 2022 to evaluate the impact of this change on key sociodemographic and smoking parameters and generally showed good comparability.^9^ However, the prevalence of exclusive non-cigarette smoking was 1.24 percentage points higher in the group surveyed via telephone than face-to-face (2.03% [95% CI = 1.42–2.90] vs. 0.79% [0.48–1.31]),^9^ suggesting the change in methodology may account for some of the increase we observed. It is not clear how far this reflects a genuine effect of mode of data collection versus natural monthly variation in responses that would not be present if the data were collected over a longer period. Because exclusive non-tobacco smoking is relatively rare, there is a high degree of variability in the estimate between months even when the modality of assessment is constant (Figure 1). In any case, our data indicate the prevalence of exclusive non-cigarette tobacco smoking is currently substantially higher than previous estimates have suggested.

A third limitation is that we used a hybrid sampling approach rather than random probability sampling. However, comparisons with other sources suggest the survey recruits a nationally representative sample and produces similar estimates of key smoking variables.^5,6^ Fourth, as a household survey, the sample excluded people living in institutions or experiencing homelessness, so our findings may not be representative of changes in exclusive non-cigarette tobacco smoking among these groups. Finally, our data do not offer any insight into use of non-combustible tobacco products (eg, heated tobacco products), which are likely to have lower risks to health,^30^ but may also be included under the proposed smoke-free generation policy.

In conclusion, while the exclusive use of non-cigarette combustible tobacco remains rare among adults in England, it has increased in recent years. As of September 2023, there were approximately 772 800 adult exclusive non-cigarette tobacco smokers in England; around five times more than in September 2013. The prevalence of exclusive non-cigarette tobacco smoking has been consistently higher among men and current vapers. The rise in prevalence has differed by age, with a more pronounced rise leading to higher prevalence in September 2023 among younger than older ages.

## Supplementary material

Supplementary material is available at *Nicotine and Tobacco Research* online.

ntae021_suppl_Supplementary_Material

## Data Availability

The data used in these analyses are available on Open Science Framework (https://osf.io/w7f3m/), with age provided in bands to preserve participant anonymity.

## References

[1] Department of Health and Social Care. Stopping the Start: Our New Plan to Create a Smokefree Generation; 2023. https://www.gov.uk/government/publications/stopping-the-start-our-new-plan-to-create-a-smokefree-generation. Accessed October 5, 2023.

[2] Li L , Borland R, Cummings KM, et al Patterns of non-cigarette tobacco and nicotine use among current cigarette smokers and recent quitters: findings from the 2020 ITC four country smoking and vaping survey. Nicotine Tob Res. 2021;23(9):1611–1616. 33693833 10.1093/ntr/ntab040 PMC8562420

[3] Office for National Statistics. Adult Smoking Habits in the UK: 2022; 2023. https://www.ons.gov.uk/peoplepopulationandcommunity/healthandsocialcare/healthandlifeexpectancies/bulletins/adultsmokinghabitsingreatbritain/2022. Accessed September 7, 2023.

[4] Ng Fat L. Health Survey for England 2015: Adult Cigarette Smoking. NHS Digital; 2016. https://files.digital.nhs.uk/publicationimport/pub22xxx/pub22610/hse2015-adult-smo.pdf. Accessed November 1, 2023.

[5] Fidler JA , Shahab L, West O, et al “The smoking toolkit study”: a national study of smoking and smoking cessation in England. BMC Public Health. 2011;11:479. doi: https://doi.org/10.1186/1471-2458-11-479 21682915 PMC3145589

[6] Jackson SE , Beard E, Kujawski B, et al Comparison of trends in self-reported cigarette consumption and sales in England, 2011 to 2018. JAMA Netw Open. 2019;2(8):e1910161. 31461148 10.1001/jamanetworkopen.2019.10161 PMC6716287

[7] Jackson SE , Beard E, Angus C, Field M, Brown J. Moderators of changes in smoking, drinking and quitting behaviour associated with the first COVID-19 lockdown in England. Addiction. 2022;117(3):772–783. 34431577 10.1111/add.15656 PMC8652848

[8] Jackson SE , Garnett C, Shahab L, Oldham M, Brown J. Association of the COVID-19 lockdown with smoking, drinking, and attempts to quit in England: an analysis of 2019-2020 data. Addiction. 2021;116(5):1233–1244. 33089562 10.1111/add.15295 PMC8436745

[9] Kock L , Tattan-Birch H, Jackson S, Shahab L, Brown J. Socio-demographic, smoking and drinking characteristics in GB: A comparison of independent telephone and face-to-face Smoking and Alcohol Toolkit surveys conducted in March 2022. Qeios. Published online August 16, 2022. doi: https://doi.org/10.32388/CLXK4D

[10] Office for National Statistics. Population estimates for the UK, England, Wales, Scotland and Northern Ireland: mid-2021. Published 2022. https://www.ons.gov.uk/peoplepopulationandcommunity/populationandmigration/populationestimates/bulletins/annualmidyearpopulationestimates/mid2021. Accessed February 7, 2023.

[11] Office for National Statistics. Annual Mid-Year Population Estimates, UK: 2013; 2014. https://www.ons.gov.uk/peoplepopulationandcommunity/populationandmigration/populationestimates/bulletins/annualmidyearpopulationestimates/2014-06-26. Accessed November 3, 2023.

[12] Vardavas CI , Nikitara K. COVID-19 and smoking: a systematic review of the evidence. Tob Induc Dis. 2020;18:20. doi: https://doi.org/10.18332/tid/119324 32206052 PMC7083240

[13] Patanavanich R , Glantz SA. Smoking is associated with COVID-19 progression: a meta-analysis. Nicotine Tob Res. 2020;22(9):1653–1656. doi: https://doi.org/10.1093/ntr/ntaa082 32399563 PMC7239135

[14] Reddy RK , Charles WN, Sklavounos A, et al The effect of smoking on COVID-19 severity: a systematic review and meta-analysis. J Med Virol. 2021;93(2):1045–1056. 32749705 10.1002/jmv.26389 PMC7436545

[15] Wackowski OA , Delnevo CD. Young adults’ risk perceptions of various tobacco products relative to cigarettes: results from the national young adult health survey. Health Educ Behav. 2016;43(3):328–336. 26304709 10.1177/1090198115599988 PMC4766060

[16] Nicola M , Alsafi Z, Sohrabi C, et al The socio-economic implications of the coronavirus pandemic (COVID-19): a review. Int J Surg. 2020;78:185–193. doi: https://doi.org/10.1016/j.ijsu.2020.04.018 32305533 PMC7162753

[17] Mehra K , Rup J, Wiese JL, et al Changes in self-reported cannabis use during the COVID-19 pandemic: a scoping review. BMC Public Health. 2023;23(1):2139. 37915021 10.1186/s12889-023-17068-7 PMC10621278

[18] UK Government. The Tobacco and Related Products Regulations 2016. Published 2016. https://www.legislation.gov.uk/uksi/2016/507/regulation/15/made. Accessed January 12, 2024.

[19] Branston JR , Hiscock R, Silver K, Arnott D, Gilmore AB. Cigarette-like cigarillo introduced to bypass taxation, standardised packaging, minimum pack sizes, and menthol ban in the UK. Tob Control. 2021;30(6):708–711. 32848080 10.1136/tobaccocontrol-2020-055700 PMC8543195

[20] Sze S , Pan D, Nevill CR, et al Ethnicity and clinical outcomes in COVID-19: a systematic review and meta-analysis. EClinicalMedicine. 2020;29:100630. doi: https://doi.org/10.1016/j.eclinm.2020.100630 33200120 PMC7658622

[21] Elliott J , Bodinier B, Whitaker M, et al COVID-19 mortality in the UK Biobank cohort: revisiting and evaluating risk factors. Eur J Epidemiol. 2021;36(3):299–309. 33587202 10.1007/s10654-021-00722-y PMC7882869

[22] Tattan-Birch H , Jackson SE, Kock L, Dockrell M, Brown J. Rapid growth in disposable e-cigarette vaping among young adults in Great Britain from 2021 to 2022: a repeat cross-sectional survey. Addiction. 2023;118(2):382–386. 36065820 10.1111/add.16044 PMC10086805

[23] Action on Smoking and Health. Use of E-Cigarettes among Adults in Great Britain; 2023. https://ash.org.uk/resources/view/use-of-e-cigarettes-among-adults-in-great-britain-2021. Accessed October 30, 2023.

[24] Action on Smoking and Health. Use of E-Cigarettes (Vapes) among Young People in Great Britain; 2023. https://ash.org.uk/resources/view/use-of-e-cigarettes-among-young-people-in-great-britain. Accessed August 17, 2023.

[25] Akl EA , Gaddam S, Gunukula SK, et al The effects of waterpipe tobacco smoking on health outcomes: a systematic review. Int J Epidemiol. 2010;39(3):834–857. 20207606 10.1093/ije/dyq002

[26] Baker F , Ainsworth SR, Dye JT, et al Health risks associated with cigar smoking. JAMA. 2000;284(6):735–740. 10927783 10.1001/jama.284.6.735

[27] Knapp H , Kirk SA. Using pencil and paper, Internet and touch-tone phones for self-administered surveys: does methodology matter? Comput Hum Behav. 2003;19(1):117–134.

[28] Rosenbaum A , Rabenhorst MM, Reddy MK, Fleming MT, Howells NL. A comparison of methods for collecting self-report data on sensitive topics. Violence Vict. 2006;21(4):461–471. 16897913

[29] Greenfield TK , Midanik LT, Rogers JD. Effects of telephone versus face-to-face interview modes on reports of alcohol consumption. Addiction. 2000;95(2):277–284. 10723856 10.1046/j.1360-0443.2000.95227714.x

[30] Tattan-Birch H , Hartmann-Boyce J, Kock L, et al Heated tobacco products for smoking cessation and reducing smoking prevalence. Cochrane Database Syst Rev. 2022;6(1). doi: https://doi.org/10.1002/14651858.CD013790.pub2 PMC8733777 34988969

